# Familial hypocalciuric hypercalcaemia type 1 caused by a novel heterozygous missense variant in the CaSR gene, p(His41Arg): two case reports

**DOI:** 10.1186/s12902-022-01231-z

**Published:** 2022-12-19

**Authors:** Aoife Courtney, Arnold Hill, Diarmuid Smith, Amar Agha

**Affiliations:** 1 Department of Endocrinology and Diabetes Mellitus, Beaumont Hospital/RCSI Medical School, Dublin, Ireland; 2 Department of General Surgery, Beaumont Hospital/RCSI Medical School, Dublin, Ireland

**Keywords:** Familial hypocalciuric hypercalcaemia, Primary hyperparathyroidism, PTH dependent hypercalcaemia, CaSR gene mutation, Calcium metabolism

## Abstract

**Background:**

Familial hypocalciuric hypercalcaemia (FHH) is a rare, inherited disorder of extracellular calcium sensing. It is clinically characterised by mild to moderate parathyroid hormone dependent hypercalcaemia, an autosomal dominant pattern of inheritance, and a normal to reduced urinary calcium excretion in spite of high serum calcium.

**Case presentation:**

We report two cases of FHH in a family caused by a novel pathogenic missense variant in the CaSR gene, p. His41Arg. Case 1, describes a 17 year old female with no significant past medical history, admitted with acute appendicitis requiring laparoscopic appendectomy and reporting a six month history of polydipsia. Routine investigations were significant for hypercalcaemia, corrected calcium 3.19 mmol/L (2.21-2.52mmol/L), elevated parathyroid hormone of 84pg/ml (15-65pg/ml) and a low 24-hour urine calcium of 0.75mmol/24 (2.50-7.50mmol/24). She was initially managed with intravenous fluids and Zolendronic acid with temporary normalisation of calcium though ultimately required commencement of Cinacalcet 30 mg daily for persistent symptomatic hypercalcaemia. Genetic analysis was subsequently positive for the above variant. Case 2, a 50-year-old female, was referred to the endocrine outpatient clinic for the management of type 2 diabetes and reported a longstanding history of asymptomatic hypercalcaemia which had not been investigated previously. Investigation revealed hypercalcaemia; corrected calcium of 2.6 mmol/L (reference range: 2.21–2.52 mmol/L); PTH of 53.7ng/L (reference range: 15–65 ng/L) and an elevated 24-hour urine calcium of 10 mmol/24 (2.50–7.50 mmol/24hr) with positive genetic analysis and is managed conservatively. Despite sharing this novel mutation, these cases have different phenotypes and their natural history is yet to be determined. Two further relatives are currently undergoing investigation for hypercalcaemia and the family have been referred for genetic counselling.

**Conclusion:**

Accurate diagnosis of FHH and differentiation from classic primary hyperparathyroidism can be challenging, however it is essential to avoid unnecessary investigations and parathyroid surgery. Genetic analysis may be helpful in establishing a diagnosis of FHH in light of the biochemical heterogeneity in this population and overlap with other causes of hypercalcaemia.

## Background

FHH is a rare, genetically heterogeneous condition clinically characterised by mild to moderate parathyroid hormone (PTH) dependent hypercalcaemia, an autosomal dominant pattern of inheritance, and normal to reduced urinary calcium excretion [1, 2]. FHH type 1 is the most common form and is caused by heterozygous loss-of-function mutations of the calcium sensing receptor (CaSR) gene [3].

The CaSR is a plasma membrane G protein coupled receptor expressed in the Chief cells of the parathyroid gland, thyroid C-cells, cells lining the renal tubules and perhaps intestinal and bone-derived cells [4, 5]. The CaSR plays a key role in mineral ion homeostasis by virtue of its role in calcium mediated PTH secretion, calcitonin secretion and renal cation handling [4, 5]. At the level of the parathyroid gland, the CaSR regulates the synthesis and secretion of PTH and parathyroid cellular proliferation [5].

Mutations in the CaSR gene result in resistance of the CaSR to extracellular calcium levels and diminished hypercalcaemia-induced calciuresis [5]. PTH levels are often normal reflecting disturbance of hypercalcaemia-induced inhibition of PTH release [5]. Patients are often asymptomatic, which may reflect resistance to the normal symptoms of hypercalcaemia [6]. Less frequently FHH results from mutations in the GNA11 and AP2S1 genes, termed FHH type 2 and 3 respectively [1, 3]. GNA11 encodes the Gα11 protein, which is involved in CaSR signalling, AP2S1 encodes AP2σ2, which plays a role in clarithin-mediated endocytosis of CaSR [1].

FHH causes lifelong hypercalcaemia, which is usually asymptomatic. When symptoms occur they are typically mild and include fatigue, weakness, and thought disturbance [2]. The phenotype is normal [7]. The frequency of urolithiasis and osteopenia are not increased, though cases of chondrocalcinosis and acute pancreatitis have been reported [2, 6].

Diagnosis of FHH can be challenging owing to the biochemical heterogeneity of the population and overlap with other causes of hypercalcaemia especially primary hyperparathyroidism, and therefore genetic analysis can be helpful in this regard [6].

## Case presentations

### Case 1

#### Case presentation

A 17 year old previously fit and well female presented to the emergency department with acute onset right iliac fossa pain, pyrexia and malaise. On examination, she was clinically hypovolemic with right iliac fossa tenderness and guarding. She was alert, orientated and there were no clinical features of endocrinopathy. Imaging was consistent with acute appendicitis and she was admitted under the care of the general surgery service for emergency laparoscopic appendectomy. Her postoperative course was complicated by COVID-19 pneumonia with persistent pyrexia and elevated inflammatory markers.

She had no significant past medical or surgical history. She was born from non-consanguineous Caucasian parents, with a normal developmental history. She denied nausea, polyuria, myalgias, arthralgias, or constipation though reported a six-month history of polydipsia drinking approximately 5 L fluid per day. Her only medication was the oral contraceptive pill. Of note, the family history was significant for three maternal cousins undergoing investigation for hypercalcaemia.

#### Investigation

Routine postoperative investigations were significant for hypercalcaemia with corrected calcium of 3.19 mmol/L (reference range: 2.21–2.52 mmol/L) and an associated elevated parathyroid hormone (PTH) of 84 ng/L (reference range: 15–65 ng/L). Phosphate 0.66mmol/L (reference range 0.81–1.45) and 25-Hydroxyvitamin D by mass spectrometry was < 10.0 nmol/L (reference range: >50 nmol/L). 24-hour urine calcium was low 0.75 mmol/24 (2.50–7.50 mmol/24hr) with a urine volume of 2425mls. Initial investigations are shown in Table 1.

**Table 1:** Case 1 initial investigation workup during inpatient stay

Investigation	Result	Reference range
Corrected Calcium (mmol/L)	3.19	2.21–2.52
Phosphate (mmol/L)	0.66	0.81–1.45
Sodium (mmol/L)	135	133–146
Potassium (mmol/L)	4.1	3.5–5.3
Creatinine (umol/L)	49	45–84
Estimated glomerular filtration rate (mL/min/1.73 m^2^)	> 60	
Parathyroid hormone (pg/ml)	84	15–65
Vitamin D total (nmol/L)	< 10.0	> 50.0
24 h urine calcium (mmol/24 hr)	1.51	2.5–7.5

Subsequently genetic analysis was performed for AP2S1, CASR and GNA11 genes as well for MEN1 and RET proto-oncogene. This was initially reported as negative for pathogenic variants. Ultrasound of the neck did not demonstrate any parathyroid or thyroid pathology and sestimibi scan showed no convincing scintigraphic evidence of parathyroid adenoma. MRI pituitary with contrast was unremarkable, which was performed because of a raised Prolactin 1010 mIU/L (reference range 102–496 mIU/L). Renal ultrasound was negative for calculi.

#### Treatment

The patient was managed with IV fluid resuscitation post appendectomy in the context of hypercalcaemia with pyrexia, COVID-19 pneumonia and significant insensible losses. However her corrected calcium failed to improve below 3 mmol/L necessitating administration of 5 mg IV Zolendronic Acid thirteen days post operatively. Corrected calcium was 2.87 mmol/L on discharge.

She was referred for ongoing endocrine follow up. Three months after discharge, the patient was symptomatic of fatigue and lethargy. Investigations were significant for a corrected Calcium of 3.08 mmol/L, Phosphate 0.67 mmol/L, PTH 57 pg/ml (reference range: 15–65 ng/L), and Vitamin D by mass spectrometry 10.7 nmol/L. These biochemical abnormalities necessitated rehospitalisation for therapeutic optimisation. During her second admission she achieved clinical and biochemical improvement with intravenous fluids and the addition of Cinacalcet 30 mg daily. She was commenced on Vitamin D when corrected calcium was controlled below 3 mmol/L. After 8 weeks of treatment the patient was asymptomatic with corrected calcium 2.45 mmol/L and PTH 16 pg/ml. The Cinacalcet dose was then reduced to 30 mg alternative days.

On subsequent follow up, corrected calcium had increased to 3.04 mmol/L and the patient reported increased polydipsia. Cinacalcet was increased back to 30 mg daily and on follow up the corrected calcium dropped to 2.8 mmol/L with an improvement in symptoms.

#### Outcome and follow up

Additional investigations were performed to assess the underlying cause of hypercalcaemia including genetic testing. Initial analysis identified a novel missense variant in the CaSR gene, p.(His41Arg), but this was not initially deemed pathogenic due to a lack of supporting evidence. This variant was later identified however in another patient with hypercalcaemia, a maternal cousin of the proband whose case is outlined later in this paper. Subsequent in silico protein modelling revealed that this variant protein is predicted to form several extra hydrogens bonds in the inactive conformation, which is likely to impair transition to the active conformation, resulting in loss of function. This variant has not been previously described to our knowledge and has not been identified in gnomAD population database. The p.His41 residue is highly conserved and has a consurf score of 8.

**Fig. 1:**
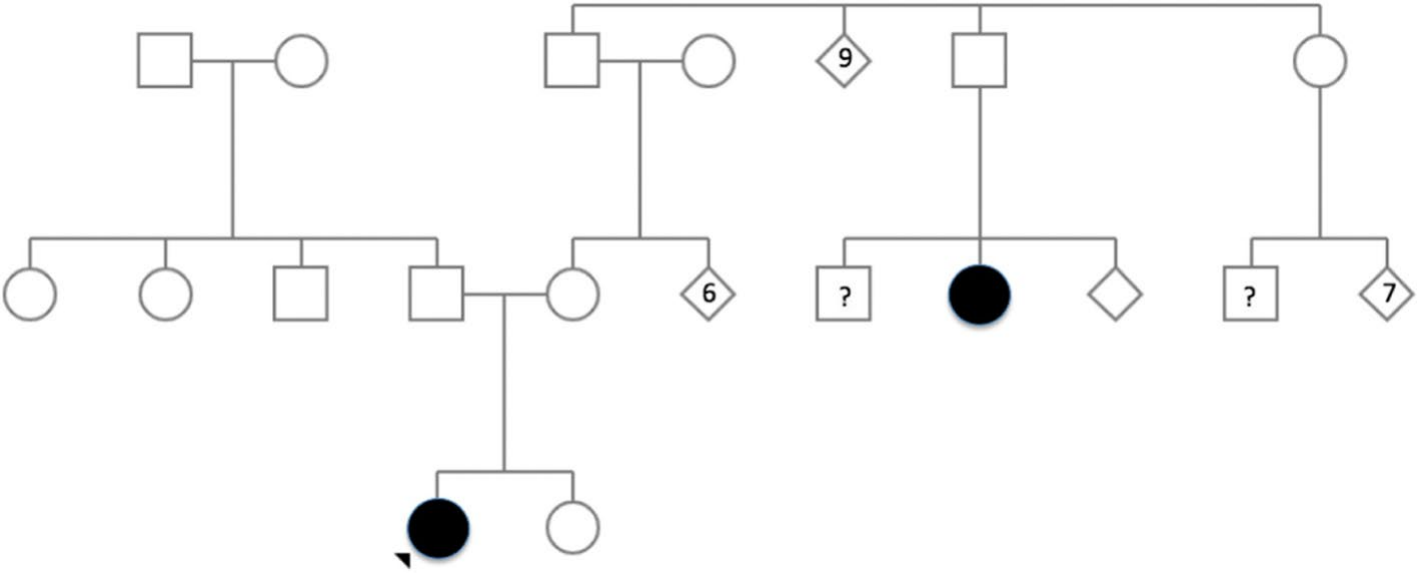
Pedigree for reported family where filled shapes represent confirmed cases of hypercalcaemia with p(His41Arg) variant detected (Cases 1 and 2). Case 1 is identified with an arrow. Individuals with a history of hypercalcaemia awaiting investigation are identified with ‘?’ symbol

In light of the above information, this variant is considered a likely pathogenic variant causing familial hypocalciuric hypercalcaemia type 1. Two further maternal cousins first removed have also since had hypercalcaemia detected on routine investigation in the community and are awaiting further investigation. A family pedigree is outlined in Fig. 1.

The patient is currently 18 years old and is taking Cinacalcet 30 mg daily. She maintains regular endocrinology surveillance at a tertiary care centre. The patient and her family have also been referred to genetic counselling services.

### Case 2

#### Case presentation

A 50-year-old female was referred to the endocrine outpatient clinic for the management of type 2 diabetes. Her past medical history was significant for type 2 diabetes, polycystic ovarian syndrome, hyperlipidaemia, obstructive sleep apnoea, a right-sided Erb’s palsy secondary to a birth injury, and epilepsy. She also reported a history of hypercalcaemia noted since early adulthood, which had never previously been investigated. Her brother and cousins also had hypercalcaemia of undetermined etiology. She reported post ictal psychosis over a 15 year period but no specific hypercalcaemic symptoms. Her medications included aripiprazole and metformin. Her examination was significant for an elevated BMI with a mildly Cushingoid appearance but otherwise unremarkable.

#### Investigations

Routine investigations were significant for a PTH dependent hypercalcaemia; corrected calcium of 2.6 mmol/L (reference range: 2.21–2.52 mmol/L) and an associated PTH of 53.7ng/L (reference range: 15–65 ng/L). 25-Hydroxyvitamin D by mass spectrometry of 31 nmol/L (reference range: >50 nmol/L); 24-hour urine calcium was 10 mmol/24 (2.50–7.50 mmol/24hr). HbA1c was 53mmol/mol (reference range 20.0–42.0 mmol/mol) and overnight dexamethasone suppression test showed normal suppression with a cortisol of 23nmol/L. On review of historical records, the highest corrected calcium in the past was 2.82mmol/L.

Ultrasound of the neck and sestamibi scintigraphy were negative for a parathyroid adenoma. A renal ultrasound showed no nephrolithiasis. Because of the family history, genetic analysis was performed for AP2S1, CASR, CDC73, CDKN1B, MEN 1 and RET genes which was significant for a heterozygous likely pathogenic CASR missense variant, p.(His41Arg), causing FHH type 1.

#### Treatment

Although her neuropsychiatric symptoms were not felt to be related to the hypercalcaemia, a trial of cinacalcet was used initially but quickly self discontinued the treatment due to gastrointestinal side effects. Vitamin D was adequately replaced with Oral Vitamin D3 800 units daily. Repeat investigations following Vitamin D replacement are outlined in Table 2. She was subsequently discharged to her GP for calcium monitoring due to non-attendance.

**Table 2:** Case 2 investigations following Vitamin D replacement

Investigation	Result	Reference range
Corrected Calcium (mmol/L)	2.68	2.1–2.6
Phosphate (mmol/L)	1.03	0.74–1.5
Sodium (mmol/L)	140	133–146
Potassium (mmol/L)	4.5	3.0–5.0
Creatinine (umol/L)	72	49.0 − 0.90.0
Estimated glomerular filtration rate (mL/min/1.73 m^2^)	74	
Parathyroid hormone (pg/ml)	40.7	15.0–68.3
Vitamin D total (nmol/L)	67	> 50.0
24 h urine calcium (mmol/24 hr)	10	2.5–7.5

## Discussion and conclusions

The genetic analysis in the CaSR gene confirmed the presence of a novel, likely pathogenic variant, c.122 A > 3 (p. His41Arg), in heterozygosity suggesting the diagnosis of FHH type 1. This variant was classified in accordance with the 2015 ACMG/AMP guidelines. Supporting evidence included in silico protein modelling, the detection of the variant in a family member with hypercalcaemia, and that the p.(His41Arg) variant is predicted by SIFT and PolyPhen to have a deleterious effect on protein function. Both our reported cases share this novel CaSR gene variant, however interestingly demonstrate significant phenotypic and biochemical differences. Case 1 presents with symptomatic severe hypercalcaemia with hypocalciuria while Case 2 presents with a milder asymptomatic hypercalcaemia and hypercalciuria. Owing to this being a novel mutation with significant heterogeneity in its phenotype we cannot be clear as to the natural course of this condition. Close observation will be required as well as identifying other affected family members.

FHH is an important differential diagnosis for hypercalcaemia. The diagnosis of FHH is suggested by a biochemical profile demonstrating the presence of mild to moderate hypercalcaemia, accompanied by a normal to high PTH level and the presence of hypocalciuria or a urinary calcium to creatinine clearance ratio of less than 0.01 [8, 9]. A calcium to creatinine clearance ratio of less than 0.01 is more than 80% and 88% specific for FHH [8].

A number of factors however may contribute to difficulties in relying on biochemical parameters alone to confirm a diagnosis. Hypocalciuria may also be caused by low dietary intake, vitamin D deficiency, renal impairment, and medications including lithium and thiazide diuretics [6, 7]. Conversely, some patients with FHH can have normal or even elevated urine calcium excretion [4]. FHH tends to run a benign course in most cases, with few if any symptoms or complications of hypercalcaemia, therefore the correct diagnosis is necessary to spare the individual and affected family members unnecessary investigation and intervention [5, 9]. Confusing the diagnosis with the more common sporadic primary hyperparathyroidism may subject patients to parathyroid surgery and its associated risks and costs when it is felt to be of little if any benefit in FHH [4, 5]. Genetic testing, as demonstrated by this case, can be useful in this regard.

In general treatment is not required in FHH [6]. Patients often experience persistent hypercalcaemia following subtotal parathyroidectomy and total parathyroidectomy results in hypoparathyrodism and as such is only rarely indicated [2]. There are however case reports of patients with FHH and concomitant parathyroid adenomas, who demonstrate improvement in serum calcium levels following surgical intervention [10–12].

Calcimimetic drugs, such as cinacalcet, are allosteric modulators at the CaSR. They increase the CaSRs affinity for Ca2 + thereby decreasing PTH concentration at any given level of Ca2+ [5, 13]. Cinacalcet is indicated for the treatment of tertiary hyperparathyroidism in adult patients with end-stage renal disease on maintenance dialysis therapy and for reduction of hypercalcaemia in adult patients with parathyroid carcinoma or primary hyperparathyroidism for whom parathyroidectomy is not appropriate.

Case reports have demonstrated clinical and biochemical improvement with Cinacalcet in some patients with symptomatic FHH due to mutations in the CASR and AP2S1 genes as well as neonatal severe hyperparathyroidism [2]. In Case 1, we also observed significant reduction in serum calcium and symptom response with Cinacalcet during short-term observation. A controlled trial would be highly desirable to further investigate the role of calcimimetics in the management of patients whose condition is not controlled with conservative management to relieve symptoms of hypercalcaemia. 

## Data Availability

Data supporting these case reports are stored locally in Beaumont Hospital, Dublin and Beaumont Private Hospital, Dublin and can be provided on request.
